# Backseat Drivers Take the Wheel

**DOI:** 10.1016/j.ccr.2007.11.020

**Published:** 2007-12-06

**Authors:** P. Andrew Futreal

**Affiliations:** 1 Cancer Genome Project, Wellcome Trust Sanger Institute, Hinxton CB10 1SA, UK

## Abstract

Somatic mutations in human cancers are comprised of those that contribute to the oncogenic phenotype, driver mutations, and those that reflect the general patterns of exposure and disrepair but are otherwise noncontributory, passenger mutations. Distinguishing drivers that can be of low frequency in any given tumor type from often more numerous passengers is a key challenge. In this issue of *Cancer Cell*, Fröhling and colleagues tackle this challenge admirably for the known cancer gene *FLT3* in acute myeloid leukemia—undertaking a systematic resequencing and functional validation approach, identifying important rare driver mutations as well as passenger mutations in patients negative for the more common activating mutations.

## Main Text

The first forays into large-scale systematic screening for somatic point mutations in human cancer have begun to reveal a heretofore undocumented complexity and diversity of human cancer at the DNA sequence level (Greenman et al., 2007; Sjöblom et al., 2006). There is growing evidence that, in addition to the more well-known culprits mutated commonly across various tumor types at appreciable prevalence, such as *TP53*, *KRAS*, and *BRAF*, there are very likely to be a substantial number of infrequently mutated cancer genes that contribute to oncogenesis. To further complicate matters (in addition to this less than ideal configuration for fully exploiting cancer gene mutations as inroads to therapeutics), the majority of somatic mutations identified in any given screen are very likely to be passenger events, with driver mutations scattered sparingly among them (Greenman et al., 2007). So, how will we sort the drivers from passengers, and what are the systematic approaches we will need to assemble to move forward and maximize translation of the information that will be coming from large-scale sequencing efforts?

The paper by Fröhling and colleagues (Fröhling et al., 2007) details one of the first systematic investigations of driver and passenger mutations incorporating comprehensive target sequencing of a substantial number of cases and functional validation of variants observed. Their work concentrates on the role of mutations in *FLT3*, a receptor tyrosine kinase, in acute myeloid leukemia (AML). Of particular note, *FLT3* mutations are already known to play an important role in AML (Small, 2006; Stirewalt and Radich, 2003). Internal tandem duplication mutations within the juxtamembrane (JM) autoinhibitory segment have been demonstrated in 25%–30% of adult AML cases (Stirewalt and Radich, 2003). Point mutations in the JM region have been show in a small number of cases. As well, activating mutations in the activation loop (AL) have been shown in an additional 7% of AML cases (Fröhling et al., 2005). Importantly, patients with *FLT3* mutant leukemias have worse prognosis with higher risks of relapse and shorter overall survival (Yanada et al., 2005).

Given the previous data, one might assume that the role of *FLT3* mutation in AML is pretty well worked out. However, the authors took a laudably systematic approach to ascertain the full spectrum of *FLT3* mutations in AML. A cohort of 222 pretreatment AML samples from adult patients negative for known activating mutations in *FLT3* (as well as *KIT* and *NRAS*) were sequenced for all coding exons and consensus splice junctions of *FLT3*. A total of 17 heterozygous nucleotide substitutions were identified. Of these, 5/17 had been previously reported as single nucleotide polymorphisms. Three of 12 unique variants were silent (synonymous) substitutions and were not pursued further; the remaining nine were missense (amino acid-changing) substitutions. Unfortunately, only one sample with a missense variant had available remission material to verify the somatic nature of the change. While the lack of confirmation of variants as somatic in any such study where variants are by definition rare is a point of concern, the specific sequence variants had not been detected in another 258 various samples where *FLT3* coding exons have been fully sequenced in other studies. Also, two affected amino acid residues, V592 and R834, have had different variants reported (http://www.sanger.ac.uk/genetics/CGP/cosmic/). The authors pragmatically treat the nine variants as “candidate” driver mutations for the purposes of moving on to functional validation.

Here is where the study comes into its own. There has been a substantial body of work put into understanding the transforming and signaling properties of mutant FTL3 proteins. In particular, the BaF3 murine hematopoietic transformation assay has proven a facile assay to detect transforming/cytokine independence-inducing mutations in a variety of *FLT3* and other tyrosine kinase alleles. Putting all nine candidate driver mutations through their paces, only four variants comprised of one extracellular domain, two JM domains, and one AL domain were able to induce IL-3-independent growth. These four transforming alleles were further shown to induce constitutive phosphorylation of FLT3 in immunoprecipitation experiments. These data suggest that these are very likely to be driver mutations. A secondary screen of an additional 127 adult AML cases for the presence of the four transforming alleles found one additional example for two of them, further supporting their identification as driver mutations.

The other five variant alleles were unable to induce growth factor independence with all cells expressing the variants undergoing apoptosis upon IL-3 withdrawal. That these variants failed to score for transformation suggests that these are likely passenger mutations. Further support to this interpretation is given by finding no further examples of the five variants in sequencing the relevant exons from 102 normal DNA samples. Also, the one proven somatic mutation in the full series is among the five putative passengers. The passenger variants were found in the extracellular domain (n = 3) and in the kinase and activation loops. Bioinformatics analysis results of all nine variants with two commonly used tools for assessing potential functional impact of amino acid substitutions (SIFT, http://blocks.fhcrc.org/sift/SIFT.html; and PMut, http://mmb2.pcb.ub.es:8080/PMut/) were a bit of a mixed bag, with only 2/4 of the transforming variants predicted to alter function of the protein. One of the putative passenger variants, G831E, although negative in the BaF3 assay, was also predicted to alter function. While there is certainly some complementary information provided by a purely bioinformatics approach, the data provide strong rationale for coupling these data with functional assays. It remains a formal possibility that the passenger alleles, in particular the G831E, which affects the highly conserved glycine in the canonical DFG motif of the AL, may have contextual oncogenic properties in the AML in which it occurred but that do not report in the BaF3 assay. As well, a number could be private rare polymorphisms. This being said, the application of a robust functional screen that reports transforming activity relevant to response to targeted therapeutics provides compelling evidence for the identification of relevant low-prevalence driver mutations and ever-present passengers.

The authors go on to show that the four driver alleles differentially activate downstream signaling pathways. The AL allele R834Q was found to only activate ERK signaling, unlike the common D835Y mutation, which activated ERK, AKT, and STAT signaling. Likewise, the two JM alleles were found to activate all three signaling pathways, while the extracellular domain allele was found to only activate ERK. These results suggest a marked subtlety in interaction of the FTL3 receptor with downstream targets modulated by the various mutations. As well, the data suggest that activation of the MAPK pathway alone via FLT3 phosphorylation and ERK activation is sufficient for transformation. Furthermore, all four driver alleles conferred sensitivity to the potent FLT3 TK inhibitor PKC412—an observation highlighting the potentially important clinical impact the identification of these alleles could have.

The work presented highlights several important points as we move forward into an era of cancer genome sequencing. First, in addition to the search for new cancer genes, we should revisit known cancer genes and fully evaluate their contribution, not only in the tumor types they are most associated with but also in a wide variety of cancers. Second, we will need robust approaches to identify relevant cancer gene mutations in patient samples in clinically meaningful timeframes. Third, the assessment of driver and passenger variants in this study was greatly facilitated by the target gene being a well-studied kinase. Applying this approach to nonkinase genes is going to be challenging and will require the development of increased throughput cell biological assay platforms. This and other systematic screens are beginning to populate our lists of cancer genes and mutations with a wide spectrum of mutation types and prevalence in an increasing variety of genes. Understanding what the forces of in vivo selection are and how they are shaping the emergence and contribution of these genes and mutations can only help us to better know the enemy, which may well be legion.
